# Incidence of asthma and mortality in a cohort of young adults: a 7-year prospective study

**DOI:** 10.1186/1465-9921-6-95

**Published:** 2005-08-16

**Authors:** Roberto de Marco, Francesca Locatelli, Lucia Cazzoletti, Massimilian Bugianio, Aurelia Carosso, Alessandra Marinoni

**Affiliations:** 1 Unit of Epidemiology and Medical Statistics, University of Verona, Verona, Italy; 2 National Health Service, CPA-ASL 4 Unit of Respiratory Medicine, Turin, Italy; 3 Department of Applied Health Sciences, University of Pavia, Pavia, Italy

## Abstract

**Background:**

Few longitudinal data exist on the incidence of asthma in young adults and on the overall mortality risk due to asthma. A 7-year follow-up prospective study was performed to assess the incidence of asthma and mortality from all causes in a cohort of young adults.

**Methods:**

The life status of a cohort of 6031 subjects, aged 20–44 years, who replied to a respiratory screening questionnaire between 1991 and 1992, was ascertained in 1999. A new questionnaire investigating the history of asthma was subsequently sent to the 5236 subjects who were still alive and residents in the areas of the study. 3880 subjects (74%) replied to the second questionnaire.

**Results:**

The incidence of adult-onset asthma was 15.3/10,000/year (95%CI:11.2–20.8). The presence of asthma-like symptoms (IRR:4.17; 95%CI:2.20–7.87) and allergic rhinitis (IRR:3.30; 95%CI:1.71–6.36) at baseline were independent predictors of the onset of asthma, which was more frequent in women (IRR:2.32; 95%CI:1.16–4.67) and increased in the younger generations.

The subjects who reported asthma attacks or nocturnal asthma symptoms at baseline had an excess mortality risk from all causes (SMR = 2.05; 95%CI:1.06–3.58) in the subsequent seven years. The excess mortality was mainly due to causes not related to respiratory diseases.

**Conclusion:**

Asthma occurrence is a relevant public health problem even in young adults. The likelihood of developing adult onset asthma is significantly higher in people suffering from allergic rhinitis, in women and in more recent generations. The presence of asthma attacks and nocturnal symptoms seems to be associated with a potential excess risk of all causes mortality.

## Introduction

Asthma is usually considered to be a chronic disease that starts in childhood, characterised by a low specific mortality risk, which can be completely avoidable with adequate management. However, recent epidemiological studies have pointed out [1] that a non negligible number of the current asthmatics in the general population developed the disease in adulthood and that adult-onset asthma has a worse prognosis than childhood asthma.

Unfortunately, few longitudinal data exist on the incidence of asthma in adults [2-4]. Consequently, knowledge of the natural history and the risk factors for adult asthma is limited and relies almost completely on prevalence data, which depend on both the incidence and the persistence of the disease. There are even fewer longitudinal studies investigating the outcome of adult asthma in terms of overall mortality [5-9], which suggest that asthma and asthma symptoms are associated with a significantly higher all-causes mortality compared to the general population.

In this paper we describe the incidence of asthma and all-causes mortality in a large, representative sample of young Italian adults, who participated in the European Respiratory Health Survey (ECRHS) stage I in 1991, and who were followed up in 1999/2000. In particular, our objective was to assess the incidence of adult onset asthma and to test : 1) whether a previous history of allergic rhinitis was a predictor for the onset of asthma, after adjusting for known risk factors; 2) whether a previous history of asthma attacks or nocturnal asthma-like symptoms was associated with a subsequent overall mortality excess in young adults.

## Methods

### i) Study design

A repeated survey of those who replied to the screening questionnaire [10] in the frame of the Italian branch of the ECRHS-stage1 in 1991/1992, was performed in three Italian centres (Verona, Pavia and Turin) during 1999/2000. The initial cohort was made up of 6031 subjects, randomly chosen from the general population. The mean age of the cohort in 1991/1992 was 32.7 years (range 20–44 years) and the percentage of women was 49.6%. All subjects were mailed a new questionnaire up to two times, in case of non response. The questionnaire administered in 1999/2000 included the same standard questions used in the first survey (enquiring about asthma attacks, wheezing, nocturnal dyspnoea, nocturnal tightness, allergic rhinitis in the last 12 months and current use of asthma drugs) with additional questions on the history of asthma (doctor diagnosis, age of the first/ last attack), the history of exposure to active smoking and social class. The questions used in the first and second surveys have been published elsewhere [11].

After the second postal wave, the life and residence status of all non responders to the mail survey were obtained from vital statistics records. Then all subjects who had neither moved nor died before or during the survey were sent a third postal wave and were finally interviewed by phone.

At the end of follow-up, 748 (12.4%) subjects had moved and 47 (0.8%) died before or during the study; 352 (5.8%) could not be traced through anagraphic records, 1004 (16.6%) did not reply or explicitly refused to answer the questionnaire and 3880 (64.3%) replied.

The protocol of the study was approved by the Italian Ethical Committees of the participating centres, and the individual information was collected in compliance with the Italian law (n°675/1996) concerning the protection of the privacy of individual health data.

### ii) Incidence analysis

5236 (86.4%) subjects from the initial cohort were considered eligible for this analysis (anyone who had moved or died was excluded). Of these, 3880 replied to the second questionnaire in 1999/2000 (response rate 74%); 24 subjects were excluded because of the mismatching of age and/or sex. The population at risk for the incidence analysis included all the asthma-free subjects in the first study (1991/92). Accordingly, 302 out of 3856 valid respondents were excluded because they were considered asthmatics at baseline: that is, people who reported having current asthma in the first study (having had an attack of asthma in the last 12 months or currently taking any medicine for asthma) as well as those who, in the second study, reported having had the first asthma attack more than 3 years before the first survey. This was done considering that a discrepancy of less than three years could be due to recall bias when reporting the age of onset (Pattaro C. Long-term repeatability of a questionnaire for lifelong asthma assessment.*Personal communication*). New cases of asthma were considered those, among the population at risk, who gave a positive answer to the question "Have you ever had asthma?" in the second survey.

Incidence rates were computed by dividing the number of new cases by the total number of person-years. PY was computed as the time between the first and second survey for non asthmatics and the time between the first interview and the first asthma attack for new asthmatics. For 6 subjects, who reported in the second survey having had the first attack of asthma less than 3 years before the first survey, the time at risk was set at 1 day [4].

Subjects, reporting in the first study, having had 'wheezing or whistling in their chest at any time' and/or having 'woken up with a feeling of tightness in the chest' and/or having 'woken up with an attack of shortness of breath' in the past 12 months, were considered as having asthma symptoms at baseline.

All subjects who reported never having smoked in the second questionnaire were considered non smokers at baseline. Subjects who reported having smoked at one time in the second questionnaire were classified as smokers or ex-smokers at baseline, according to their answers to the following questions: 'How old were you when you started smoking?' and 'How old were you when you stopped smoking?'.

According to postal codes, each subject was considered a resident either in urban or suburban areas. Areas were classified as suburban when the inhabitants of the municipal district were less than 40,000, urban otherwise. Participants were divided into two social classes based on their profession: low (blue collars/unemployed/retired) and medium/high (manager/white collars/teachers...).

The Poisson regression model [12] was used to assess the association of baseline symptoms and individual characteristics with the incidence of asthma.

Statistical analysis was performed using STATA software [13], release 7.0 (Stata Corp 1997. Stata Statistical Software: release 5.0. Stata Corporation, College Station, TX).

### iii) Mortality analysis

All subjects were considered eligible for this analysis. Mortality rates were computed by dividing the number of deaths during the follow-up by the person-years (PY) at risk [12]. PY were computed as: i) the time between the first and the second survey (follow-up time) for responders to the questionnaire; ii) the average follow-up time for non responders; iii) the time between the first survey and the date of death for deceased people; iv) half follow-up time for subjects who had moved before the second survey or were untraceable (censored alive at mid follow-up). Mortality rates were estimated according to symptoms reported at baseline. Standardised Mortality Ratios (SMR adjusted by age, sex and centre) were used to compare the mortality rates in subjects with and without symptoms at baseline.

The specific cause of death was ascertained using the mortality records of the health districts of the three centres involved in the survey; the underlying cause of death was coded according to the International Classification of Diseases, Ninth Revision. The specific cause of death was not found for one subject who had died outside the health district. As information on smoking habits was missing in the first ECRHS questionnaire, it was not possible to adjust the SMR for this variable. However, to verify the stability of our results, a sensitivity analysis was performed on all the subjects who replied to the second questionnaire (3856) and who had the information on smoking habits. In this subgroup, SMRs adjusted for smoking habits were computed assuming that the 47 deaths were 1) all non smokers; 2) all smokers; 3) had the same smoking distribution of the respondents (random assignment).

## Results

### Incidence

**Figure 1:**
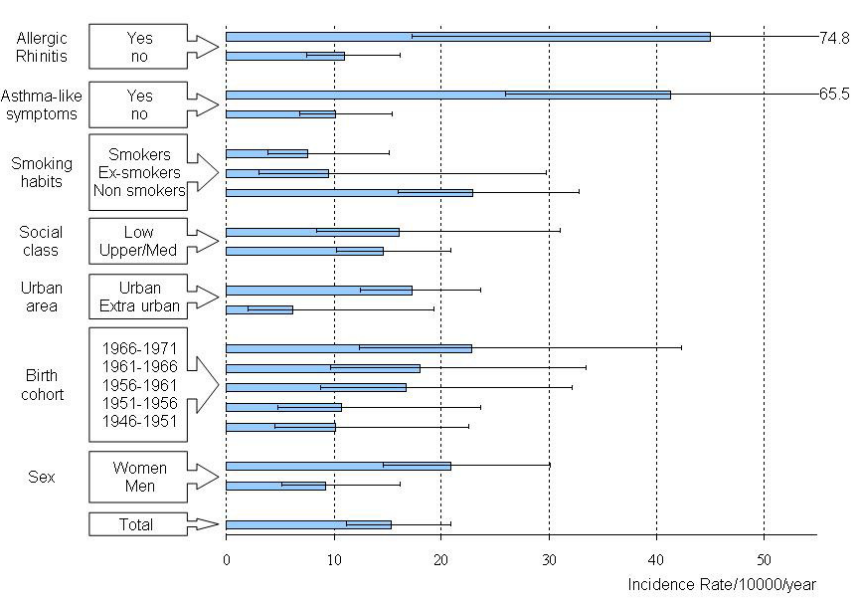
Average annual incidence of asthma (*10,000/year) and 95% confidence interval during the period 1991–2000, according to baseline characteristics (reported in the first study:1991/1992).

Subjects who were included in the incidence analysis were found to be slightly older (33.1 vs 32.5 years, p < 0.05) and there was a greater percentage of women (51.2% vs 47.8%, p < 0.005) than non responders to the second questionnaire; however, there was no statistically significant difference in symptoms at baseline (first study) between responders and non responders. The average time of follow-up for the incidence study was 7.72 years. Forty-one new cases of asthma out of 3554 people at risk occurred during the period between the two studies (Table 1), and the average annual incidence rate was 15.2/10000/year (95%CI: 11.2–20.7), ranging from 10.1/10000/year in the older generation to 22.8/10000/year in the younger generation. The incidence rate for asthma was greater in women (Figure 1) than men, higher in the younger groups and in urban than suburban areas, lower in active smokers than non smokers and peaked in subjects who reported asthma-like symptoms (wheezing and/or nocturnal dyspnoea and/or nocturnal tightness in the chest) and allergic rhinitis at baseline.

**Table 1:** Number of subjects at risk at start of follow-up (1991/92), number of new cases of asthma, person-years, crude incidence (95% C.I.) during the period 1991–2000 according to sex and birth cohort.

	N° of subjects at risk	N° of new cases (1991–2000)	Person-years	Incidence*10,000
**Total**	3554	41	26881	15.25 (11.23–20.71)
				
**Sex**				
Men	1720	12	13040	9.20 (5.23–16.20)
Women	1834	29	13841	20.95 (14.56–30.15)
				
**Birth Cohort**				
1946–1951	793	6	5935	10.11 (4.54–22.50)
1951–1956	738	6	5634	10.65 (4.78–23.71)
1956–1961	710	9	5379	16.73 (8.70–32.15)
1961–1966	732	10	5543	18.04 (9.71–33.52)
1966–1971	581	10	4389	22.78 (12.26–42.34)

After adjusting for all these factors, the incidence of asthma (Table 2) was significantly and independently associated with sex (Incidence rate ratio, IRR = 2.32, 95%CI:1.16–4.67), the presence of asthma-like symptoms (IRR = 4.17, 95%CI: 2.20–7.87) and allergic rhinitis (IRR = 3.30, 95%CI: 1.71–6.36); it was also significantly lower in smokers than non-smokers (IRR = 0.33; 95%CI:0.15–0.73). The incidence of asthma showed a significantly increasing trend (p < 0.01) according to the birth cohort.

**Table 2:** Mutually Adjusted Incidence Rate Ratios (95% C.I.) for the association of the incidence of asthma with the main baseline characteristics (1991/1992).

	**Relative risk (IRR)**	**95% C.I.**	**p-value**
**Sex**			
Men	1		
Women	2.32	1.16–4.67	0.017
			
Birth cohort *			
1946–1951	1		
1951–1956	1.12	0.36–3.49	0.84
1956–1961	1.69	0.60–4.77	0.32
1961–1976	1.68	0.60–4.69	0.32
1966–1971	2.28	0.82–6.34	0.11
			
**Urban area**			
Suburban area	1		
Urban area	2.52	0.77–8.19	0.12
			
Asthma-like symptoms**			
No	1		
Yes	4.17	2.20–7.87	<0.001
			
**Allergic rhinitis**			
No	1		
Yes	3.30	1.71–6.36	<0.001
			
**Smoking habits**			
Non smokers	1		
Ex-smokers	0.49	0.15–1.65	0.25
Smokers	0.33	0.15–0.73	<0.01
			
**Social class**			
Medium /High	1		
Low	1.65	0.77–3.52	0.20

* test for trend p ≤ 0.01

** wheezing and/or nocturnal shortness of breath and/or nocturnal tightness in the chest

### Mortality

Average follow-up time for the mortality study was 7.05 years. Forty-seven subjects from the initial cohort died between 1991 and 2000. The average annual mortality rate was 11.0/10000/year (95%CI: 8.3–14.7), consistent with the official mortality data for the same age groups and the same areas (Table 3). Mortality rates were particularly high (Table 4) for people reporting having had at least one attack of asthma (32.2/10000/year), nocturnal dyspnoea (30.3/10000/year) and nocturnal tightness (23.5/10000/year). Subjects who reported no respiratory symptoms at baseline had very similar mortality rates to those who reported wheezing, allergic rhinitis or nocturnal cough.

**Table 3:** Number of deaths, person-years, average annual mortality rates (95% Confidence Interval) for the ECRHS-Italy cohort (1991–2000),and official mortality rates in northern Italy.

	Number of deaths	Person-years	Mortality rate *10,000 (95% C.I.)	Northern Italy mortality rates *10,000 ^$^
Total	47	42523	11.0 (8.3–14.7)	10.9
				
Sex				
Men	34	21592	15.7 (11.2–22.0)	14.7
Women	13	20931	6.2 (3.6–10.7)	6.9

^$ ^ISTAT (National Institute of statistics) 1998

**Table 4:** Number of subjects who reported a specific symptom in the first survey (1991/1992), number of deaths observed during the follow-up (1991–2000), crude annual mortality rate (*10,000/year), and its 95% Confidence Interval.

	Number of subjects at risk	Deaths during 1991–2000	Annual mortality rate*10,000	95% Confidence Interval
No respiratory symptoms	3225	26	11.4	7.7–16.7
Wheezing	600	4	9.4	3.5–25.1
Allergic rhinitis	959	7	10.5	5.0–22.1
Nocturnal Cough	1771	10	8.0	4.3–14.8
Nocturnal Tightness	1771	8	23.5	11.7–46.9
Nocturnal Dyspnoea	429	9	30.3	15.8–58.3
Asthma attacks	221	5	32.2	13.4–77.3

The reporting of asthma attacks and/or nocturnal dyspnoea and/or nocturnal tightness in the first survey (1991/92) was associated with a two-fold increase in subsequent overall mortality (SMR = 2.05; 95%CI:1.06–3.58) compared to the rest of the population, after adjusting for age, sex and centre (Table 5). The increase in overall mortality for this group of subjects was mainly due to a statistically significant increase in mortality from accidents and a moderate increase in mortality from cancer and cardiovascular diseases. No deaths from respiratory diseases were observed in our cohort, while 4 out of 16 deaths from tumours were due to lung cancer (1 in the symptomatic group and 3 in the control group). When the one death with unknown cause was attributed to each specific cause of death, the interpretation of the results was the same (see last column of Table 5).

**Table 5:** Number of deaths (1991–2000), average annual mortality rates for overall and specific causes of death in subjects with or without asthma attacks/nocturnal dyspnoea/nocturnal tightness (A/D/T) in the first survey (1991/1992) and Standardized Mortality Ratios adjusted for sex, age and centre.

	Subjects without A/D/T (n = 5169)		Subjects with A/D/T (n = 862)			
			
	Number of deaths	Crude mortality rate *10,000	Number of deaths	Crude mortality rate *10,000	SMR (95% C.I.) ^$^	Simulated SMR (95% C.I.)^$$^
**All causes**	35	9.58	12	19.96	2.05 (1.06–3.58)	2.05 (1.06–3.58)
All tumours (140–239)	12	*3.29*	4	*6.65*	2.09 (0.57–5.36)	1.80 (0.49–4.61)
- Lung cancer (160–165)	3	*0.82*	1	*1.66*	2.01 (0.05–11.18)	1.53 (0.04–6.52)
Cardiovascular diseases (390–459)	4	*1.10*	1	*1.66*	1.34 (0.03–7.47)	1.11 (0.03–6.18)
Accidents (800–999)	4	*1.10*	4	*6.65*	6.95 (1.89–17.80)	5.47 (1.49–14.01)
Other causes	14	*3.83*	3	*4.99*	1.29 (0.27–3.78)	1.21 (0.25–3.54)
Unknown	1	*0.27*	0	*0.0*	0.00	

^$ ^with respect to subjects without A/D/T

$$ SMR was computed attributing the one death with unknown cause to each of the specific causes of death

The sensitivity analysis confirmed the stability of the previous results. In fact, in the subgroup of subjects with smoking information, the SMR not adjusted for smoking was 1.99 (95CI:1.03–3.48) while those adjusted for smoking were: 1.91 assuming that all the deaths were smokers; 2.02 assuming that all the deaths were non smokers and 1.94 when the same distribution of smoking among the responders was assumed.

The mortality rates for subjects with asthma attacks/nocturnal symptoms and reporting to be or not to be under treatment were 14.4/10000/year (95% C.I.: 2.0–102.3) and 20.9/10000/year (95% C.I.: 11.6–37.8), respectively, and were not statistically different (p = 0.72).

## Discussion

Our results provide information about incidence of adult asthma and all causes mortality risk related to asthma in northern Italy, an area with a relatively low prevalence of asthma [14]. The main findings of our longitudinal analysis were as follows:

i) The average incidence of adult onset-asthma in northern Italy is 15.2/10,000/year, which ranks in the lower part of the ranges reported in longitudinal studies in other countries. Allergic rhinitis and asthma-like symptoms are strong and independent predictors of adult-onset asthma, which occurs more frequently in women and in recent generations.

ii) The presence of asthma attacks and nocturnal asthma symptoms (tightness and dyspnoea) in young adults is associated with a two-fold increase in the risk of dying in the subsequent seven years, compared to asymptomatic subjects.

### Incidence

In our analysis of the incidence of asthma, the population at risk and the new cases of the disease were defined according to the occurrence or absence of a relevant clinical event, such as an asthma attack, which is less likely to be influenced by recall bias and more reproducible than the reporting of asthma symptoms [15,16]. It has also been reported that, at least in Italy, the large majority (85%) of subjects who self-reported an attack of asthma had been diagnosed by a doctor [1]. Consequently, it is likely that our definition slightly underestimates the incidence of the disease compared to symptom-based definition [17] on one hand; but then on the other, it guarantees better specificity [18] and stability of our estimates than other definitions.

Our estimate of the average annual incidence of asthma in young adults in Italy was 15.2/10,000/year, ranging from 10.1/10,000/year, in the older generations, to 22.8/10,000/year in the younger generations. Our longitudinal study confirms that adult asthma occurs more frequently in women and in younger generations. The gender difference in the occurrence of asthma has been pointed out in several previous studies [17,19,20] from different countries and it has been attributed mainly to the role of female sex hormones [21]. The reasons for the generalized increase in asthma incidence in the youngest generations are still unknown. Changes in susceptibility to environmental stimuli leading to asthma (hygiene hypothesis) as well as changes in exposure to environmental factors (increase in air pollution) could have contributed to this increase [22,23]. Our finding that incidence of asthma is higher in urban than suburban areas support both the previous hypotheses.

The strongest independent predictor of the incidence of asthma was the presence of asthma-like symptoms, such as diurnal wheezing and nocturnal dyspnoea and tightness, in agreement with a recent longitudinal Spanish study [4]. This finding indicates that there is a group of subjects for which the clinical phase of the disease starts with mild symptoms which are probably not recognized as asthma, but which will be diagnosed in the following years when the disease gets worse and the exacerbations are labelled as asthma attacks in the end.

The presence of allergic rhinitis at baseline, regardless of other asthma symptoms, was another strong predictor of asthma. This result concords with other longitudinal studies showing that allergic rhinitis is an independent risk factor for the onset of asthma [24,2].

The association between allergic rhinitis and asthma has been traditionally interpreted as the progression of a common airway disease associated with atopy [25,26]. It has also been reported [24,27,28] that rhinitis is a significant risk factor for adult asthma in both atopic and non atopic subjects, suggesting that other mechanisms, besides atopy, should be invoked to explain the observed association [29,30]. Our study, based on questionnaire data and not on a measurement of atopy, cannot contribute to highlight this hypotheses. However, the strong association between early allergic rhinitis and onset of asthma in adulthood suggests that earlier treatment of allergic rhinitis symptoms [31,32] could modify the natural evolution of asthma and prevent the development of a more severe disease. Indeed, interventions such immunotherapy [33], pharmacologic therapy [34] and allergens avoidance [35] for allergic rhinitis seem to be effective in preventing complications and the development of asthma [29-31].

In agreement with other longitudinal studies [36,37], we found that active smoking does not increase the risk of asthma in adults, probably because susceptible people simply do not start smoking or quit smoking very quickly. This finding is in contrast with another longitudinal study on adolescents [38], where active smoking was a risk factor for the incidence of asthma. In any case, whether active smoking is a risk factor for the incidence of asthma or whether it is a selection factor (healthy smoker effect) continues to be an open problem.

Finally, in contrast to a recent paper [39] that found an association between asthma prevalence and socioeconomic status, our results do not support that being in a low social class increases the risk of developing asthma. This suggest that previously reported associations, based on prevalence studies, reflect more the association of social class with the severity and/or the persistence of the disease rather than its association with the occurrence of asthma.

### Mortality

To our knowledge, few studies have been published on the overall mortality risks of adults with asthma or asthma-like symptoms. This is one of the few, that prospectively investigated asthma related mortality risk in a large cohort of young adults. We ascertained life status for all subjects still resident (82%) in the same areas where they lived in 1991, while we assumed that people who were untraceable or who had moved before the second survey were censored alive halfway through the follow-up. As there are no reasons to expect that censoring could be related to the outcome or presence of asthma symptoms, our estimates of the mortality risk are not biased by potential selection in the initial cohort.

The presence of asthma attacks and/or nocturnal asthma symptoms was associated with an increased mortality risk from all causes (SMR = 2.05; 95% CI:1.06–3.58). Our estimate of the relative mortality risk for asthmatics agrees with those previously reported, which ranged from 1.5 [9] to 2.4 [5].

Although the excess death due to chronic obstructive diseases accounted for a substantial part of the mortality risk in other studies [9,5,6], in our study it was mainly due to non respiratory causes of death, such as accidents, and to a lesser extent, to cardiovascular diseases and cancer, which concords with a French study [8]. This discrepancy can be explained by the fact that our cohort included much younger individuals than previous studies, and it is well known that young people < 45 years are at a much lesser risk of dying from chronic diseases than older people.

Death from accidents is the leading cause of death in the general population aged 15–44 years, at least in Italy. Our results showed that asthmatics belonging to this age group had a 5- to 6-fold risk of dying from accidents than the general population; this suggests that a previous history of asthma attacks and nocturnal symptoms made them even more susceptible to this kind of hazard. This could be due to the fact that asthmatic symptoms, especially nocturnal ones, may interfere with diurnal attentiveness, which could cause fatal occupational accidents [40] as well as car accidents [41]. Furthermore, it has been reported that asthma patients have high rates of anxiety disorders and major depression [42,43], which can also make them more prone to accidents. Another aspect to take into consideration is the fact that many asthmatic subjects also take antihistamines for their symptoms [45], which could have a sedative effect [46] and alter their diurnal concentration. However, in our study, no association was found between the use of drugs reported in the first study and subsequent mortality.

The moderate increase in mortality from cardiovascular diseases and cancer has already been reported [44,9] and could reflect the greater susceptibility of asthmatics to traditional risk factors, which could be fully appreciated in cohorts older than ours. No association was found between wheezing at baseline and mortality likely because this symptom alone has either a low specificity for asthma [18] or identifies a very mild form of the disease. Furthermore, nocturnal asthma-like symptoms or asthma attacks could be partially attributable to other diseases like psychiatric illnesses and heart diseases.

As the screening questionnaire used in the first ECRHS study did not include information on smoking habits, it was impossible to adjust for this factor in the mortality analysis. However, the sensitivity analysis carried out on a sizable sample showed that when smoking information was taken into account, the results were very similar, suggesting that the failure to adjust for this potential confounder could have biased our results only to a minor extent.

Nevertheless, some caveat in the interpretation of our mortality results should be taken into account due to the relatively short follow-up period (7 years) and the consequent low number of deaths. As a consequence, our analysis cannot establish a definite causal relationship between the presence of severe asthma symptoms and the subsequent overall mortality due to both the observational nature of our survey and the expected low number of deaths. The absence of a specific asthma related risk of death in our young cohort does not weaken our result: in fact, mortality from asthma is completely avoidable in this age group, and no deaths are expected to be found if health services are adequate [47]. Consequently, the suggestion emerging from our study of an increased non specific mortality risk even in young subjects reporting severe asthma symptoms should be considered as a warning signal, indicating that asthma symptoms may involve a more general risk than normally expected, and should at least promote other studies dealing with the overall mortality pattern in asthmatics.

## Conclusion

The incidence of asthma is a relevant public health problem even in young adults. The likelihood of developing adult onset asthma is significantly higher in people suffering from allergic rhinitis and mild asthma-like symptoms, in women and in more recent generations. Furthermore, our results suggest that the presence of asthma attacks and nocturnal symptoms may be associated with an excess risk of all causes mortality. Greater medical attention should be paid to early asthma-like symptoms (particularly nocturnal ones) and allergic rhinitis.

## List of Abbreviations

ECRHS: European Community Respiratory Health Survey

PY: Person-years

SMR: Standardised Mortality Ratio

IRR: Incidence Rate Ratio

## Competing interests

RdeM has received a reimbursement for travel expenses to the ERS congress by GlaxoSmithKline Italia in 2003 and 2004.

## Authors' contributions

RdM developed the idea for the study and was responsible for the study design.

FL was responsible for the data management and analysis.

All the authors were responsible for the data collection in local centres and participated in the interpretation and presentation of the results.

All authors read and approved the final manuscript.
